# Stress Signals During Sucking Activity Are Associated With Longer Transition Time to Full Oral Feeding in Premature Infants

**DOI:** 10.3389/fped.2018.00054

**Published:** 2018-03-12

**Authors:** You Gyoung Yi, Byung-Mo Oh, Seung Han Shin, Jin Yong Shin, Ee-Kyung Kim, Hyung-Ik Shin

**Affiliations:** ^1^Department of Rehabilitation Medicine, Seoul National University Hospital, Seoul National University College of Medicine, Seoul, South Korea; ^2^Division of Neonatology, Department of Pediatrics, Seoul National University Children’s Hospital, Seoul, South Korea; ^3^Department of Pediatric Occupational Therapy, Seoul National University Children’s Hospital, Seoul, South Korea

**Keywords:** premature infant, feeding behavior, Neonatal Oral-Motor Assessment Scale, feeding difficulty, incoordination

## Abstract

Several treatments have been proposed to shorten the time to the attainment of full oral feeding (FOF) for premature infants, but there are only a few evaluation methods useful in estimating predictors of this period. We investigated whether specific items within the disorganized sucking patterns described by the Neonatal Oral-Motor Assessment Scale (NOMAS) could estimate the time to FOF in preterm infants with feeding difficulty. Preterm infants diagnosed with a disorganized sucking pattern in the NOMAS evaluation before 50 weeks of postmenstrual age were included. Video recordings of at least 2 min of oral feeding were further analyzed retrospectively by two assessors and the premature infants who exhibited disorganized sucking patterns (*n* = 109) were divided into three clusters (clusters 2–4). The observational items compatible with disorganization in the original NOMAS were divided into three groups: cluster 2 (disorganized: arrhythmical), cluster 3 (disorganized: arrhythmical + unable to sustain), and cluster 4 (disorganized: arrhythmical + incoordination ± unable to sustain) and further divided into incoordination-positive (cluster 4) and incoordination-negative groups (clusters 2 and 3). Premature infants in the incoordination-positive group (cluster 4, which means stress signals) showed a median transition time of 22 days (range: 4–121 days) which was longer than that in the incoordination-negative group (median 6 days; range: 1–25 days). Univariate linear regression analysis revealed that the presence of incoordination among disorganized sucking patterns (NOMAS cluster 4 vs. clusters 2 and 3), birth weight, total parenteral nutrition (TPN) duration, non-invasive positive pressure ventilation duration, the presence of moderate to severe bronchopulmonary dysplasia, pulmonary hypertension, sepsis, small for gestational age (SGA), and necrotizing enterocolitis are associated with the transition time to FOF. In a multivariate linear regression analysis, the variables revealed to be associated with the transition time were TPN duration, SGA, and the presence of stress signals (incoordination-positive group) among disorganized sucking patterns. When selecting premature infants to be treated with swallowing therapy, it is reasonable to pay more attention to the incoordination-positive group described in the NOMAS, that is, premature infants with stress signals to shorten the time to attain FOF.

## Introduction

Achieving full oral feeding (FOF) early in preterm infants can shorten hospitalization time, reduce hospital costs, and enable greater interaction between the mother and child (1–3). Strategies, such as non-nutritive sucking using a pacifier, sensorimotor stimulation, and actively pacing suck–feeds, have been used to facilitate suck–swallow function and have been reported to be effective in reducing the time to reach FOF in premature infants (2, 4–10). Therefore, at the moment of transition from tube feeding to oral feeding, predicting the time to reach FOF could be useful in choosing candidates who may require additional facilitation techniques.

The normal sucking pattern is the coordination of suck, swallow, and respiration (SSR), which means that the jaw and tongue make rhythmic movements. The term “feeding difficulty” is used to express the problem of swallowing in the broad sense, including all states that do not allow FOF. If the abnormality of oromotor function is thought to be the cause of feeding difficulty, the evaluation is often performed to distinguish between the disorganized sucking pattern and the dysfunctional sucking pattern to interpret feeding difficulty (11–13). The disorganized sucking pattern refers to a lack of rhythm of the total sucking activity. If jaw and tongue movements are abnormal resulting the interruption of the feeding process, they are defined as dysfunctional sucking patterns, which is generally known to reflect neuromuscular dysfunction.

The Neonatal Oral-Motor Assessment Scale (NOMAS) is a method of visual observation that is used to assess non-nutritive and nutritive sucking in an infant from birth to 48 weeks of postmenstrual age (PMA) (11, 12). The NOMAS consists of 28 items: 14 related to movements of the tongue and 14 related to movements of the jaw. Both movements are classified into three groups: normal, disorganization, and dysfunction (11–13). da Costa et al. reported that incoordination, under disorganization on the NOMAS, was more prevalent in preterm infants with bronchopulmonary dysplasia (BPD) than those without BPD (14). They further suggest that successful feeding is hindered by decreases in oxygen saturation during feeding, which deprives preterm infants of essential sensory and motor experiences (2, 15), leading to the late achievement of FOF.

The observational items compatible with disorganization in the original NOMAS were divided into three groups according to the presence of arrhythmical, unable to sustain, and the incoordination items (16): cluster 2 (disorganized: arrhythmical), 3 (disorganized: arrhythmical + unable to sustain), and 4 (disorganized: arrhythmical + incoordination ± unable to sustain) (Table 1). Although this cluster system grouped various NOMAS findings into categories, the clinical usefulness of each item (arrhythmical, unable to sustain, and incoordination) has yet to be demonstrated.

**Table 1:** Scoring instructions and interpretation for each Neonatal Oral-Motor Assessment Scale cluster.

Cluster	Interpretation	Scoring instruction
1	Normal sucking pattern	
2	Disorganized sucking pattern	Only an arrhythmical sucking pattern, without the observation of “unable to sustain” or “incoordination of suck/swallow and respiration” sucking patterns
3	Disorganized sucking pattern	An arrhythmical and “unable to sustain” suckle patternThe “unable to sustain” suckle pattern includes the following: The infant ceases sucking completely during the first 2 min of nutritive sucking, or The pauses are longer than the burst, or The bursts are shorter than three sucking phases
4	Disorganized sucking pattern	An arrhythmical and “incoordination of suck/swallow and respiration” sucking patterns that cause stress signals; the “unable to sustain” suckle pattern may or may not be present“Incoordination of suck/swallow and respiration” includes all the following stress signals: nasal flaring, head turning, head bobbing, extraneous movements of the body or limbs, gagging, choking, coughing, yelping, and grunting
5	Dysfunctional sucking pattern	The interruption of sucking activity owing to abnormal movements of the tongue and jaw which includes the following: Excessively wide excursions of the jaw or Minimal excursions: clenching or Flaccid tongue with absent tongue groove or Retracted tongue with posterior humping

Observational findings of the “incoordination of SSR that results in stress signals” in the NOMAS include nasal flaring, head turning, and extraneous movements of the body or limbs during sucking, as the description in the study by Palmer et al. (17). The NOMAS working group in the Netherlands added further stress signs, such as choking, gagging, coughing, yelping, and grunting, to the findings of incoordination, and grouped these symptoms as cluster 4 (16). As the authors designated, these signs are essentially stress signals, and it is still unclear whether these clinical symptoms develop owing to lack of coordination in SSR.

In this study, we investigated whether the stress signals during bottle feeding were associated with a longer time to FOF in preterm infants who showed feeding difficulty at the beginning of the oral feeding.

## Materials and Methods

### Subjects

Between May 2014 and March 2017, 148 infants in Neonatal Intensive Care Unit (NICU) were referred for consultation to the Division of Pediatric Rehabilitation for feeding difficulty during the transition period from enteral tube feeding to oral feeding. Regarding all infants who were referred, a video recording was done for over 2 min, which was a necessary preparation for the NOMAS evaluation (12, 16). Infants were eligible for inclusion if they were born preterm (<37 weeks), video recordings of oral feeding were obtained for more than 2 min for the NOMAS evaluation before 50 weeks of PMA, and if they were diagnosed with a disorganized sucking pattern in NOMAS. Premature infants were excluded if the NOMAS assessment point was more than 72 h after oral feeding initiation or if the infant received postnatal surgery resulting in the interruption of oral feeding. The result of the NOMAS evaluation was integrated into the electronic medical record, which the authors analyzed retrospectively. Medical records, as well as video recordings, were retrospectively reviewed by the authors.

All procedures performed in studies involving human participants were in accordance with the ethical standards of the institutional and/or national research committee and with the 1964 Helsinki declaration and its later amendments or comparable ethical standards. Ethical approval was obtained from the Seoul National University Hospital Institutional Review Board (No. 1705-122-855).

### Clinical Characteristics

The following parameters were investigated by retrospective medical records analysis: birth weight (BW), gestational age (GA) at birth, sex, Apgar score at 1 and 5 min after birth, total parenteral nutrition (TPN) duration, and PMA at the time of the NOMAS assessment (Table 2). We also investigated the history of BPD, germinal matrix hemorrhage, intraventricular hemorrhage (IVH), periventricular leukomalacia, invasive ventilator use after birth, the duration of non-invasive positive pressure ventilation (NIPPV) including high-flow nasal cannula and nasal continuous positive airway pressure, small for gestational age (SGA), sepsis, necrotizing enterocolitis (NEC), respiratory distress syndrome (RDS), and pulmonary hypertension (Table 2).

**Table 2:** Subjects’ characteristics with disorganized sucking pattern in the NOMAS.

Characteristic	Total (*n* = 109)	Incoordination-negative group (*n* = 77)	Incoordination-positive group (*n* = 32)	*p*-Value
GA at birth (weeks)	29.86 (2.92)	30.51 (2.68)	28.39 (2.99)	<0.001
Female/male	57/52	42/35	15/17	0.465
Birthweight (g)	1.27 [0.42, 3.05]	1.3 [0.48, 3.05]	1.02 [0.42, 2.45]	0.002
TPN duration (days)	9 [0, 73]	7 [0, 59]	17 [0, 73]	<0.001
Days on non-invasive ventilation	18 [0, 88]	12 [0, 75]	36 [0, 88]	<0.001
PMA at NOMAS evaluation (weeks)	34 + 3 [29, 49 + 4]	34 + 4 [32, 42 + 6]	34 + 2 [29, 49 + 4]	0.984
Apgar score (1 min)	4 [0, 9]	5 [0, 9]	3 [0, 7]	0.001
Apgar score (5 min)	7 [0, 10]	7 [0, 10]	6 [2, 9]	<0.001
Moderate to severe BPD	27 (24.8)	14 (18.18)	13 (40.63)	0.020
Ultrasonic finding, *n* (%)				0.593
Normal				
Grade 1–2	52 (47.71)	42 (54.55)	10 (31.25)	
GMH or IVH	52 (47.71)	32 (41.56)	20 (62.50)	
Grade 3–4				
GMH or IVH	5 (4.59)	3 (3.90)	2 (6.25)	
Invasive ventilator use	56 (51.38)	32 (41.56)	24 (75.0)	0.001
Sepsis	8 (7.34)	1 (1.30)	7 (21.88)	<0.001
Necrotizing enterocolitis	4 (3.67)	0 (0)	4 (12.5)	0.002
Respiratory distress syndrome	75 (68.81)	49 (63.64)	26 (81.25)	0.071
Small for gestational age	13 (11.93)	8 (10.39)	5 (15.63)	0.442
Pulmonary hypertension	7 (6.42)	4 (5.19)	3 (9.38)	0.418

*Continuous variables following the normal distribution are denoted by mean (SD), while continuous variables, which did not follow the normal distribution, are denoted by Median [range]. Categorical variables are denoted by *n* (%)*.

*GA, gestational age; TPN, total parenteral nutrition; PMA, postmenstrual age; NOMAS, Neonatal Oral-Motor Assessment Scale; BPD, bronchopulmonary dysplasia; GMH, germinal matrix hemorrhage; IVH, intraventricular hemorrhage*.

### Neonatal Oral-Motor Assessment Scale

Regarding all infants who were referred, a video recording of bottle feeding was done for over 2 min, which was a necessary preparation for the NOMAS evaluation. The caloric density of breast milk was variable and known to be about 57–65 kcal/100 ml (18). Premature infant powdered milk had a calorie density of 75–85 kcal/100 ml, depending on the preparation concentration (19). Video recordings within 72 h of commencement was assessed using the NOMAS (12) by a rehabilitation doctor (one of the coauthors of this paper) who has been certified by Marjorie Meyer Palmer, the original developer of the NOMAS. Video recordings included a close-up lateral view of the mouth, jaw, and neck, and only the bottle feeding was recorded. The videotaping was initiated before the lips reached the bottle nipple, and the recording was stopped after more than 2 min of the oral feeding had occurred. During the recordings, feeding was performed by an NICU nurse. To investigate interrater reliability at the cluster level, an occupational therapist, who had more than 5 years of experiences in oromotor and swallowing training for infants admitted to the NICU, also blindly assessed the NOMAS cluster using the same video recordings. The first rater (rehabilitation doctor) was blinded to clinical factors but not to GA and PMA, and the second rater (an occupational therapist) was blinded to all clinical factors.

Findings from the 28 items in the NOMAS were categorized into five clusters according to the suggestions of the Dutch NOMAS working group. An arrhythmic sucking pattern in isolation was classified as cluster 2. The NOMAS cluster 3 was defined as adding the “unable to sustain” item to cluster 2, which was defined by at least one of the following findings: (1) the infant stops sucking completely in the first 2 min of nutritive sucking; (2) the pause is longer than the burst; or (3) the bursts are shorter than three sucks. Cluster 4 refers to the addition of at least one of the “incoordination” items to cluster 2 or 3. Incoordination items defined by the Dutch group include stress signals, such as head bobbing, extraneous movements of the body or limbs during sucking, choking, gagging, coughing, yelping, and grunting (16). Disorganized sucking patterns were further divided into incoordination-positive (cluster 4, which means stress signals) and incoordination-negative groups (clusters 2 and 3).

### Outcome Measures

The primary outcome was the transition time from the initiation of oral feeding (IOF) to FOF according to the presence of the incoordination item among the disorganized sucking patterns. FOF was defined as the ability to feed three or more times only by bottle feeding, which corresponds to the time of removing the nasogastric tube (15).

### Statistical Analysis

We analyzed the data from the presence of the stress signals (incoordination-positive vs. incoordination-negative group, cluster 4 vs. clusters 2 and 3) among disorganized sucking patterns in terms of transition time to FOF and baseline characteristics. The continuous variables, including transition time, were compared between the two groups (cluster 4 vs. clusters 2 and 3) using the Mann–Whitney *U* test. For the analysis of the categorical variables, the chi-square test or Fisher’s exact test was performed. A *p*-value < 0.05 was considered statistically significant. Cohen’s kappa was obtained for the interrater reliability of the two evaluators in the cluster level and the presence of the incoordination item.

Univariate linear regression analysis was performed with the transition time to FOF as the dependent variable. Afterward, the multivariate linear regression was carried out through a stepwise selection (entry condition *p* < 0.05, removal condition *p* > 0.15). Analyses were performed using the IBM SPSS 20 software (IBM Corporation, New York, NY, USA).

## Results

### Interrater Reliability for NOMAS Clustering

The two evaluators agreed on the cluster level for 103 out of the 109 recordings (Cohen’s κ = 0.825). Disagreement occurred between clusters 3 and 4 (three infants) and clusters 2 and 3 (three infants). The reliability of the two evaluators on the presence of the incoordination items (cluster 4 vs. cluster 2 or 3) was higher with the Cohen’s κ of 0.933, with only three disagreements occurring.

### Subjects

**Figure 1:**
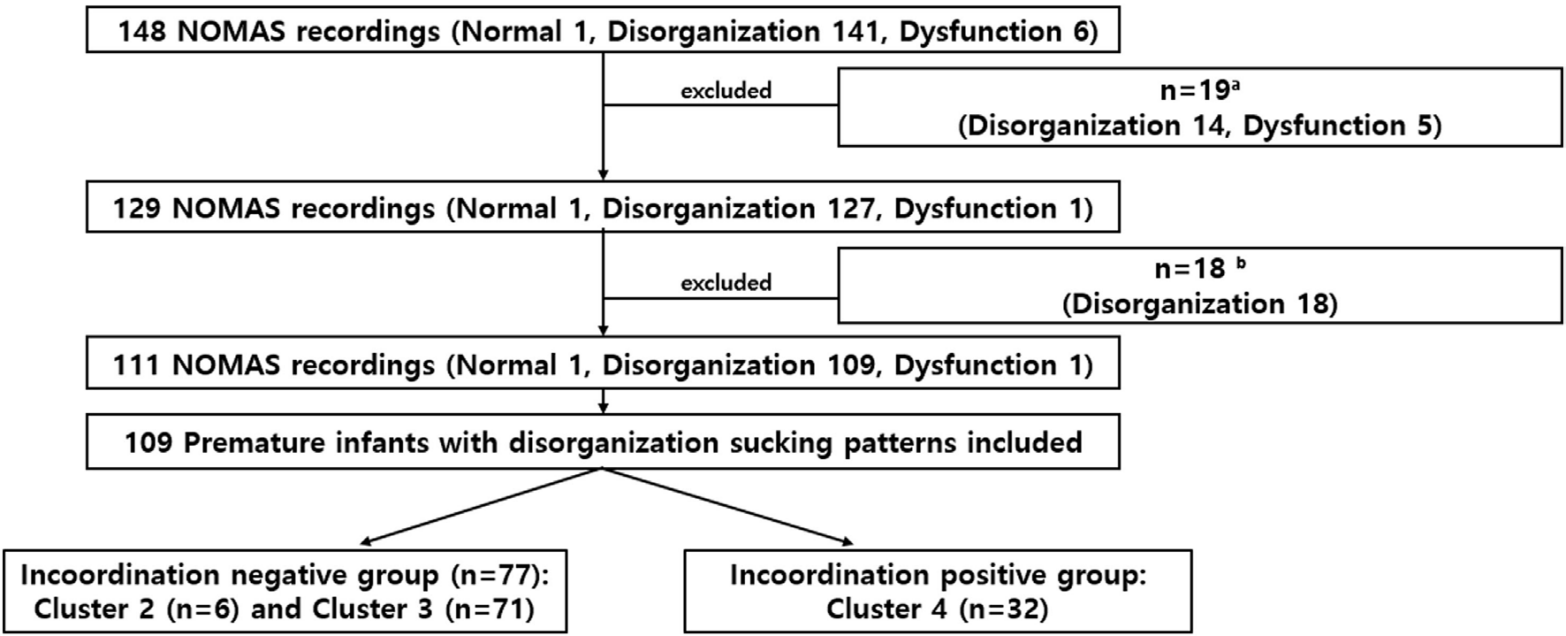
Study flowchart. Of the 148 infants evaluated, time to full oral feeding was compared in 109 preterm infants with disorganized sucking patterns. ^a^Excluded if assessment of the NOMAS was performed at >postmenstrual age 50 weeks or GA ≥ 37 weeks.^b^ Excluded if the infant received postnatal surgery resulting in the interruption of oral feeding (*n* = 6) or the NOMAS assessment point was later than 72 h after oral feeding initiation (*n* = 12).

From 148 infants with the NOMAS assessments, 14 infants with oromotor disorganization (lack of rhythm of total sucking activity), and 5 infants with oromotor dysfunction (interruption in the feeding process due to abnormal movements of the tongue and jaw) were excluded because the PMA at the time of the NOMAS assessment exceeded 50 weeks, or because GA exceeded 37 weeks. Of the remaining 129 NOMAS evaluation records, 18 cases were further excluded because the infants received postnatal surgery resulting in the interruption of oral feeding (*n* = 6) or because the NOMAS assessment point was more than 72 h after oral feeding initiation (*n* = 12). Of these 111 preterm infants, 109 preterm infants belonged to the disorganization group and were included in our study: incoordination-negative group (clusters 2 and 3) and incoordination-positive group (cluster 4, presence of stress signals) (Figure 1).

### Clinical Characteristics

The baseline characteristics and the differences according to the presence of an incoordination item (stress signals) among the disorganized sucking patterns are summarized in Table 2. Subjects in the incoordination-positive group, showed a younger GA, and a lower BW compared with those in the incoordination-negative group, among the disorganized sucking pattern preterm infants. However, there were no differences between these two groups in PMA during the NOMAS evaluation.

### Time to FOF According to Presence of Incoordination Item Among Disorganized Sucking

**Figure 2:**
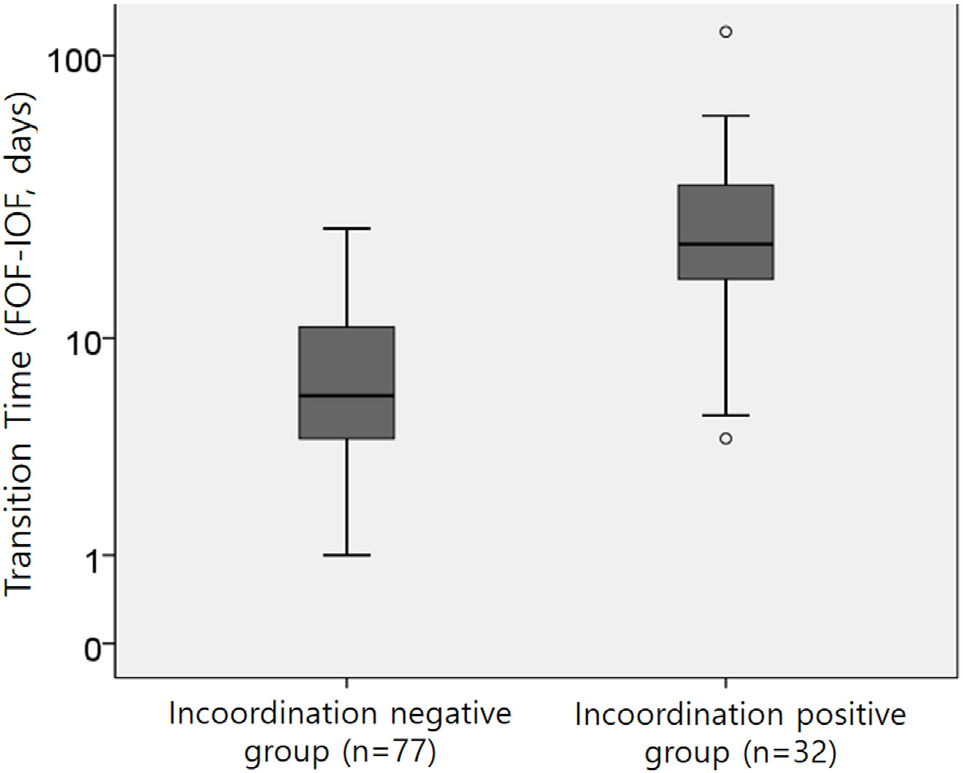
The transition time from the initiation of oral feeding (IOF) to full oral feeding (FOF) between the two groups; *p* < 0.001 by the Mann–Whitney *U* test.

The median [range] transition time to FOF of 109 preterm infants was 9 days [1–121 days]. As shown in Figure 2, there were differences in the transition time to FOF between the incoordination-positive and -negative groups. The incoordination-positive group (cluster 4, presence of stress signals) had a longer transition time of 22 days [4–121 days] compared with those of the incoordination-negative group (cluster 2 or 3, median 6 days; range: 1–25 days).

### Factors Related to Transition Time to FOF

A univariate analysis was performed to estimate factors related to the transition time (Table 3). In the univariate analysis, the presence of incoordination items (NOMAS cluster 4 vs. clusters 2 and 3), BW, TPN duration, NIPPV duration and the presence of moderate to severe BPD, pulmonary hypertension, sepsis, SGA, and NEC were determined to represent factors affecting the transition time. In the multivariate analysis, the *R* value of the final model was 0.699, and the included variables were TPN duration, SGA, and the presence of incoordination items (NOMAS cluster 4 vs. clusters 2 and 3) (Table 4).

**Table 3:** Univariate linear regression analysis for the transition time to FOF in preterm infants with disorganized sucking patterns.

Variable	*B*	95% CI for *B*		*p*-Value
Presence of stress signals (NOMAS cluster 4 vs. clusters 2 and 3)	19.351	16.704	21.998	<0.0001
Male vs. female	1.890	−3.958	7.738	0.523
Gestational age	−0.131	−0.273	0.010	0.069
Birth weight	−9.187	−14.790	−3.584	0.002
TPN duration	0.626	0.443	0.809	<0.0001
NIPPV duration	0.309	0.188	0.431	<0.0001
Moderate/severe BPD vs. none/mild BPD	11.454	5.040	17.868	0.001
Grade 3/4 GMH, IVH vs. none, grade 1/2 GMH, IVH	0.165	−6.891	7.221	0.981
Sepsis yes vs. no	21.835	11.423	32.248	<0.0001
NEC yes vs. no	10.233	−5.208	25.674	0.192
RDS yes vs. no	4.481	−1.777	10.739	0.159
SGA yes vs. no	12.546	3.843	21.250	0.005
Pulmonary hypertension yes vs. no	13.817	2.176	25.457	0.021
Apgar 1	−0.690	−1.995	0.614	0.297
Apgar 5	−1.209	−2.565	0.148	0.080

*FOF, full oral feeding; NOMAS, Neonatal Oral-Motor Assessment Scale; BPD, bronchopulmonary dysplasia; TPN, total parenteral nutrition; NIPPV, non-invasive positive pressure ventilation; GMH, germinal matrix hemorrhage; IVH, intraventricular hemorrhage; NEC, necrotizing enterocolitis; RDS, respiratory distress syndrome; SGA, small for gestational age*.

**Table 4:** Multiple linear regression analysis for transition time to FOF in preterm infants with feeding difficulty.

Variable	*B*	95% CI for*B*	Beta	*t*	*p*-Value
Presence of stress signals (NOMAS cluster 4 vs. clusters 2 and 3)	14.063	11.507–16.619	0.419	7.310	<0.001
TPN duration (days)	0.407	0.320–0.494	0.357	4.658	<0.001
SGA yes vs. no	9.065	2.048–16.082	0.192	2.562	0.012

*FOF, full oral feeding; NOMAS, Neonatal Oral-Motor Assessment Scale; TPN, Total parenteral nutrition; SGA, small for gestational age*.

## Discussion

In this study, premature infants with feeding difficulties corresponding to NOMAS cluster 4, which means the presence of stress signals (Arrhythmical + incoordination ± unable to sustain), showed a longer transit time to FOF than did those of the incoordination-negative group (clusters 2 and 3).

An interesting finding is that patients with SSR coordination reached early FOF quicker than did those with incoordination. For successful oral feeding, premature infants should achieve maturity with developments in the central and enteric nervous system-mediated reflexes involved in the activation, control, coordination, and adaptation of oromotor, aerodigestive, and peristaltic functions (20–22). In coordination-positive patients, these reflexes might be activated in the critical window of the maturational timeline, leading them to ultimately reach early FOF. These findings suggest that preservation of neuromuscular and pattern generator functions could be augmented with feeding therapies. Clusters 2 and 3 may also require therapy, but the transition time is much shorter than cluster 4, which is likely to be less prominent in terms of reducing the transition time.

The stress signals observed in the premature infants may be due to incomplete maturation of the aerodigestive system. Jadcherla et al. reported that swallow-integrated esophageal motility is an important factor in distinguishing between a primary oral feeder and a chronic tube feeder (21). Swallow frequency, swallow propagation, presence of adaptive peristaltic reflexes, oral feeding challenge test results, and upper esophageal sphincter tone were also reported to be important factors in feeding outcomes (21), demonstrating that swallowing is a complex task that requires close regulation and coordination between aerodigestive reflexes and respiratory status. The regulation of swallowing and aerodigestive pathways involves vagal and supranuclear neural pathways and is influenced by perinatal events, prematurity, and inflammatory state. Therefore, if stress signals are observed in a premature infant, the period until FOF is considered to be somewhat delayed.

Although there might be a relationship between the incoordination of the SSR and the NOMAS cluster 4 (stress signals regarded as the results from incoordination of SSR), the meaning of these two items is different. Therefore, the results of this study could be interpreted as follows: the stress signals in the NOMAS during nutritive sucking in preterm infants are more associated with a longer transition time. Therefore, if sucking patterns in premature infants are compatible with the NOMAS cluster 4 (stress signals regarded as the results from incoordination of SSR), active oromotor facilitation and swallowing training, such as applying pacifier, non-nutritive oral-motor stimulation, massage therapy, and direct tactile stimulation on specific oral structures, could be considered as supplemental oral sensory and motor experiences, and as a result, shorten the transition time to FOF. Also, giving the infant time for maturation could be considered.

Neonatal Oral-Motor Assessment Scale defines incoordination as an observational finding, while Lau et al. objectively evaluate these findings through a catheter with a pressure sensor (23). During oral feeding, respiration is disrupted, resulting in reduced ventilation and tidal volume and increased apneic episodes. This change in breathing is not caused by sucking alone but also by the interruption of airflow during swallowing. Lau et al. (24, 25) quantitatively measured nutritive sucking (suction and expression), swallowing events (as identified by hyoid upward movement), and respiration to simultaneously monitor the SSR. In their study, suction was defined as the intraoral negative pressure that draws liquid into the mouth and the expression is defined as compression and/or stripping of the tongue against the hard palate to eject liquid into the mouth. By contrast, NOMAS has the advantage of being able to evaluate these factors without specific measuring equipment and with high interrater reliability among incoordination items.

Lau and Smith (26) also evaluated fatigue and endurance by measuring the proficiency (PRO, % volume taken during the first 5 min/total volume prescribed) and the rate of milk transfer (RT, ml/min) during the entire feeding event. They reported that there are four different levels of oral feeding skills, with different feeding duration and transition time depending on the level. Although this approach has the advantage of being able to conduct evaluations through the entire duration of the feeding event, it may not reflect the intermittent stress signs of the infant during feeding, nor is it possible to distinguish between unable to sustain (cluster 3) and incoordination (cluster 4) items.

Previous studies have reported that GA, BW, and medical conditions, such as BPD, cardiac, gastrointestinal, and neurological conditions were factors which could affect the time to reach FOF (15, 17, 27–29). However, there is a distinct paucity of research targeting the oromotor function itself as a predictive factor for time to FOF.

Bingham et al. reported that the NOMAS was a poor predictor (30) and noted that feeding efficiency, such as volume consumed in the first 5 min of bottle feeding, consumption rate (ml/min), and other baseline traits were better predictors of feeding skills in premature infants. The authors in the study used subscores of the NOMAS which consisted of 12 dichotomous assessments of nutritive suck organization (suck rhythm) and 8 assessments of nutritive suck function (lip seal, tongue, and jaw movement). However, without selecting critical items such as incoordination, they counted the number of items checked in the NOMAS, which might have influenced their results. In their study, the dysfunction subscore did not change over time, but the disorganization score improved. In contrast, our present results showed that specific items, especially when an incoordination item is observed among the disorganized sucking pattern, were a strong predictor in the multivariate models. In this study, only preterm infants with disorganization sucking patterns were included, with all of these infants successfully reaching FOF.

Jadcherla et al. (31) reported an aerodigestive protective mechanism of respiratory support in infants with severe BPD and reported delayed feeding milestones, including longer gavage feeding duration in the non-invasive respiratory support group than in the control group. Notably, incoordination was more frequent in patients with longer NIPPV duration in this study, as is shown in Table 2. The potential reason for this finding is that the severity of BPD was different between the two groups or the NIPPV duration may be shortened due to early surfactant administration in the group with a coordinated sucking pattern.

There have been inconsistent results from studies regarding the implication of feeding difficulty on the neurodevelopmental outcomes in preterm infants (32–34). This might have at least partially contributed to the differences in interpreting the findings of the NOMAS. Some authors simply divided the NOMAS findings into three groups: normal, disorganization, and dysfunction (32–34), while others summed up entire items, which were checked dichotomously (30). Since these interpretive methods have still not been established as being useful in predicting the neurodevelopmental outcome, the presence of incoordination, defined by the Dutch group, could be another option for interpreting the findings of the NOMAS and utilizing this scale in an appropriate manner. Further studies are needed to identify which items of the NOMAS represent a delayed attainment of the maturational process or are indicative of neurological dysfunction.

### Study Limitations

There are some limitations to this study. Since instrumental evaluation was not included and measurements were only made through video recordings, it is possible that the process of sucking was not objectively evaluated. If manometry, pulse oximetry, and plethysmography had been added through a multidisciplinary approach, a more integrated interpretation might have been possible. This study examined the time to reach FOF from the IOF but did not evaluate the long-term feeding performance or neurodevelopment outcome of preterm infants. In this study, the stress signals of the incoordination-positive group included head bobbing, extraneous movements of the body or limbs, choking, gagging, coughing, yelping, and grunting. However, each stress signal is thought to be different. For future studies, incoordination symptoms could be further classified into different subcategories and investigated for their relative implications upon oral feeding and development.

## Conclusion

The stress signals reflecting incoordination of SSR among disorganized sucking pattern are associated with the transition time to FOF in preterm infants with feeding difficulties. When selecting premature infants to be treated with swallowing therapy, it is reasonable to perform treatment to shorten the time to attain FOF with an incoordination-positive group in the NOMAS, that is, premature infants with stress signals.

## Ethics Statement

All procedures performed in studies involving human participants were in accordance with the ethical standards of the institutional and/or national research committee and with the 1964 Helsinki declaration and its later amendments or comparable ethical standards. Ethical approval was obtained from the Seoul National University Hospital Institutional Review Board (IRB) No. 1705-122-855.

## Author Contributions

YY: acquisition of data; analysis and interpretation of data; analysis and interpretation of data; and critical revision of manuscript. B-MO: acquisition of data; study concept and design. SS: acquisition of data; study concept and design. JS: acquisition of data. E-KK: acquisition of data; study concept and design. H-IS: study concept and design; acquisition of data; analysis and interpretation of data; study supervision; and critical revision of manuscript for intellectual content.

## Conflict of Interest Statement

The authors declare that the research was conducted in the absence of any commercial or financial relationships that could be construed as a potential conflict of interest.
